# Regulation of Translocator Protein 18 kDa (TSPO) Expression in Rat and Human Male Germ Cells

**DOI:** 10.3390/ijms17091486

**Published:** 2016-09-06

**Authors:** Gurpreet Manku, Martine Culty

**Affiliations:** 1 The Research Institute of the McGill University Health Centre, McGill University, Montreal, QC H4A 3J1, Canada; gurpreet.manku@mail.mcgill.ca; 2 Departments of Medicine, McGill University, Montreal, QC H4A 3J1, Canada; 3 Pharmacology & Therapeutics, McGill University, Montreal, QC H3G 1Y6, Canada

**Keywords:** gonocytes, spermatogonia, translocator protein 18 kDa (TSPO), differentiation, human testis, germ cells, seminoma

## Abstract

Translocator protein 18 kDa (TSPO) is a high affinity cholesterol- and drug-binding protein highly expressed in steroidogenic cells, such as Leydig cells, where it plays a role in cholesterol mitochondrial transport. We have previously shown that TSPO is expressed in postnatal day 3 rat gonocytes, precursors of spermatogonial stem cells. Gonocytes undergo regulated phases of proliferation and migration, followed by retinoic acid (RA)-induced differentiation. Understanding these processes is important since their disruption may lead to the formation of carcinoma in situ, a precursor of testicular germ cell tumors (TGCTs). Previously, we showed that TSPO ligands do not regulate gonocyte proliferation. In the present study, we found that TSPO expression is downregulated in differentiating gonocytes. Similarly, in F9 embryonal carcinoma cells, a mouse TGCT cell line with embryonic stem cell properties, there is a significant decrease in TSPO expression during RA-induced differentiation. Silencing TSPO expression in gonocytes increased the stimulatory effect of RA on the expression of the differentiation marker *Stra8*, suggesting that TSPO exerts a repressive role on differentiation. Furthermore, in normal human testes, TSPO was located not only in Leydig cells, but also in discrete spermatogenic phases such as the forming acrosome of round spermatids. By contrast, seminomas, the most common type of TGCT, presented high levels of TSPO mRNA. TSPO protein was expressed in the cytoplasmic compartment of seminoma cells, identified by their nuclear expression of the transcription factors OCT4 and AP2G. Thus, TSPO appears to be tightly regulated during germ cell differentiation, and to be deregulated in seminomas, suggesting a role in germ cell development and pathology.

## 1. Introduction

Translocator protein 18 kDa (TSPO; previously known as peripheral benzodiazepine receptor PBR) is a high affinity drug- and cholesterol-binding protein strongly expressed in steroidogenic cells, including testicular Leydig cells, where it is located at the outer mitochondrial membrane (OMM) [1,2,3]. TSPO plays an essential role in cholesterol transfer from the outer to the inner mitochondrial membrane, where CYP11A1 catalyzes pregnenolone formation from cholesterol, the first step of the steroidogenic cascade [1]. Although present mainly as monomers, TSPO can form polymers upon hormonal stimulation and oxidative stress, and is also found as polymers in tumorigenic cells [2,4]. In addition to steroidogenic tissues, TSPO is expressed in multiple tissues and cells, where it is involved in various processes, including cellular proliferation, apoptosis, transport, and differentiation [4,5,6,7]. While the role of TSPO in steroidogenic cells has been well documented, its role in non-steroidogenic cells is not well understood.

We previously made the serendipitous observation that TSPO was present in neonatal rat gonocytes (also known as pre- and pro-spermatogonia), the precursor cells of type A spermatogonia, including cells of the first spermatogenic wave and spermatogonial stem cells (SSCs) [8]. In gonocytes, TSPO expression was nuclear, and its drug ligand did not regulate gonocyte proliferation [8]. TSPO was also found in pachytene spermatocytes and dividing spermatogonia in adult rat testis [8]. In a recent study, we found that TSPO transcripts are also abundant in adult mouse spermatogonia, pachytene spermatocytes and round spermatids, while the protein is observed in mouse sperm [9].

Adequate gonocyte development is critical for the establishment of a SSC reservoir that will support the production of sperm throughout adulthood [10]. These transitional cells undergo proliferation and migration to the basement membrane of the seminiferous cords, prior to their differentiation [10,11,12]. We have previously shown that gonocyte proliferation is stimulated by the coordinated action of platelet-derived growth factor (PDGF)-BB and 17β-estradiol, and requires ERK1/2 pathway activation [13,14]. We also found that gonocyte differentiation is stimulated by all-trans retinoic acid (RA) and involves the activation of PDGFR, SRC and JAK2/STAT5 pathways, highlighting the complexity of this process [15,16]. Another important regulatory process is the apoptosis of gonocytes that failed to migrate and/or differentiate, taking place at the end of the first postnatal week [17]. Studies have suggested that improper gonocyte development may lead to infertility and testicular germ cell tumors (TGCTs), a reproductive pathology on the rise in the past decades [18,19]. Thus, better understanding of gonocyte development should elucidate the mechanisms leading to germline stem cell formation, as well as the origins of TGCTs.

In the present study, we examined whether TSPO is altered during differentiation in gonocytes and in the F9 embryonal carcinoma cell line, a mouse TGCT that has retained embryonic stem cell properties, as well as somatic and germ cell markers [16]. TSPO’s potential role in RA-induced gonocyte differentiation was examined by silencing its expression. TSPO expression profiles were also determined in human adult normal testis and TGCTs. Together, these studies suggest that TSPO is regulated during germ cell differentiation and in germ cell tumors.

## 2. Results

### 2.1. Changes in TSPO Expression during Gonocyte Differentiation and in Spermatogonia

**Figure 1:**
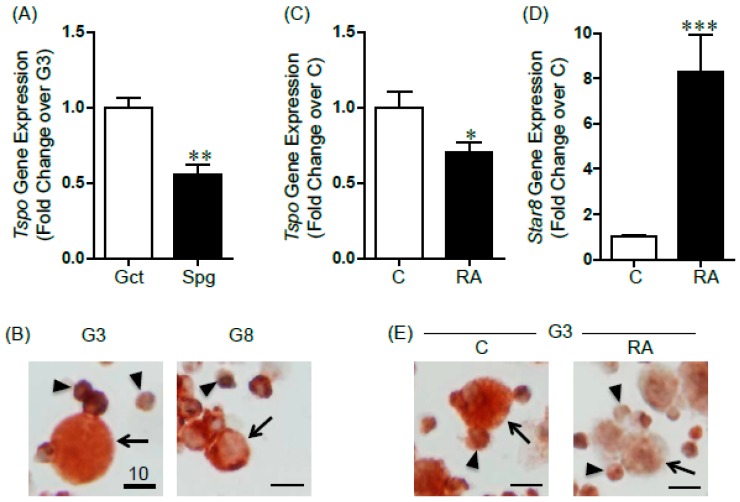
Translocator protein 18 kDa (TSPO) expression in differentiating rat gonocytes and in spermatogonia. (**A**) *Tspo* mRNA expression was determined by quantitative-PCR (qPCR) analysis in high purity PND3 gonocytes (Gct) and PND8 spermatogonia (Spg). Results shown are from 3–4 independent germ cell preparations and are plotted as mean ± SEM. ** *p*-value < 0.01; (**B**) immunocytochemical (ICC) analysis of TSPO protein expression in low purity isolated PND3 gonocytes (G3) and PND8 spermatogonia (G8). Arrows indicate germ cells; arrowheads indicate somatic cells. Scale bar = 10 μm; (**C**,**D**) mRNA expression of *Tspo* (**C**) and *Stra8* (**D**) in gonocytes treated with or without retinoic acid (RA, 10^−6^ M) for 24 h in medium supplemented with 2.5% FBS. Results shown are from 5 independent germ cell preparations with each treatment done in duplicate wells, and are plotted as mean ± SEM. * *p*-value < 0.05; *** *p*-value < 0.001; (**E**) ICC analysis of TSPO protein expression in low purity isolated gonocytes treated with or without RA (10^−6^ M) for 72 h. Arrows, arrowheads and scales are as in (**B**). (**B**,**E**) Representative cells are shown.

We have previously shown that TSPO is expressed in the nucleus of PND3 gonocytes but that binding of its drug ligand does not affect gonocyte proliferation. Here, the comparison of *Tspo* mRNA levels between purified PND3 gonocytes and the more differentiated PND8 spermatogonia showed that gonocytes expressed significantly higher amounts of *Tspo* compared to spermatogonia (Figure 1A). TSPO protein levels were also higher in gonocytes, where it appeared nuclear, than in spermatogonia, where it was found in the cytoplasm (Figure 1B). At both ages, the low purity cell preparations contained somatic cells that were either TSPO-positive (likely contaminant Leydig cells) or TSPO-negative (likely myoid and Sertoli cells) (Figure 1B).

We then treated isolated gonocytes with RA, previously shown to induces gonocyte differentiation [15,16], and found that RA treatment induced a significant downregulation (30% decrease) of *Tspo* mRNA expression, concomitant with an eight-fold increase in the differentiation marker *Stra8* (Figure 1C,D). TSPO protein levels were also decreased in RA-treated gonocytes (Figure 1E). These data suggested that TSPO is actively down-regulated during gonocyte differentiation.

### 2.2. TSPO Expression in Differentiating F9 Mouse Embryonal Carcinoma Cells

**Figure 2:**
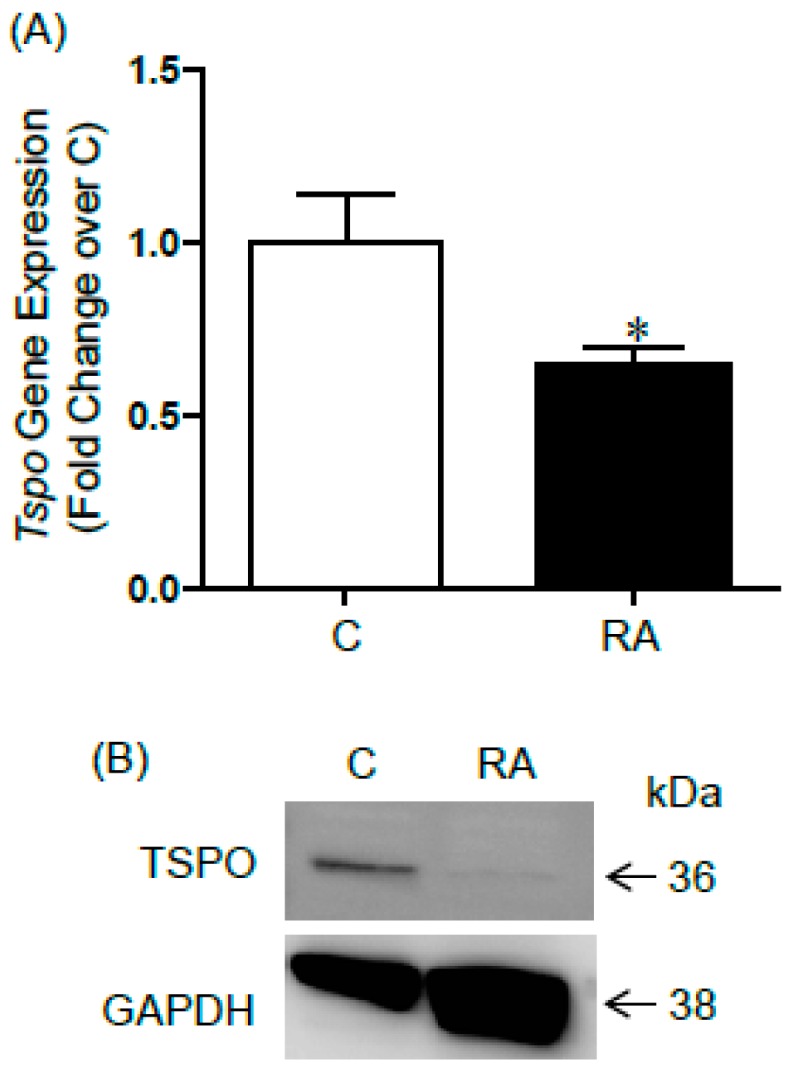
Changes in TSPO expression in differentiating F9 embryonal carcinoma cells. (**A**) *Tspo* mRNA expression in F9 cells treated with or without RA (10^−7^ M) for 24 h in medium supplemented with 10% FBS. Results shown are from 5 independent experiments, with each treatment done in duplicate wells, and are plotted as mean ± SEM. * *p*-value < 0.05; (**B**) immunoblot analysis of TSPO protein expression in F9 cells treated with or without RA (10^−7^ M) for 72 h. TSPO 36 kDa dimer band is observed. GAPDH: Loading control. A representative immunoblot is shown.

We have previously used F9 embryonal carcinoma cells as a model to study signalling pathways involved in RA-induced differentiation [15,16]. Here, we found that TSPO mRNA expression was significantly down-regulated in F9 cells treated with RA, similarly to what was observed in gonocytes (Figure 2A). TSPO protein, which was mainly found as 36 kDa dimers in F9 cells, was also greatly depleted in RA-treated F9 cells, suggesting an active down-regulation of TSPO during F9 cell differentiation (Figure 2B).

### 2.3. Effects of TSPO Knockdown on Gonocyte Differentiation and on the Expression of Germ Cell Specific Genes

**Figure 3:**
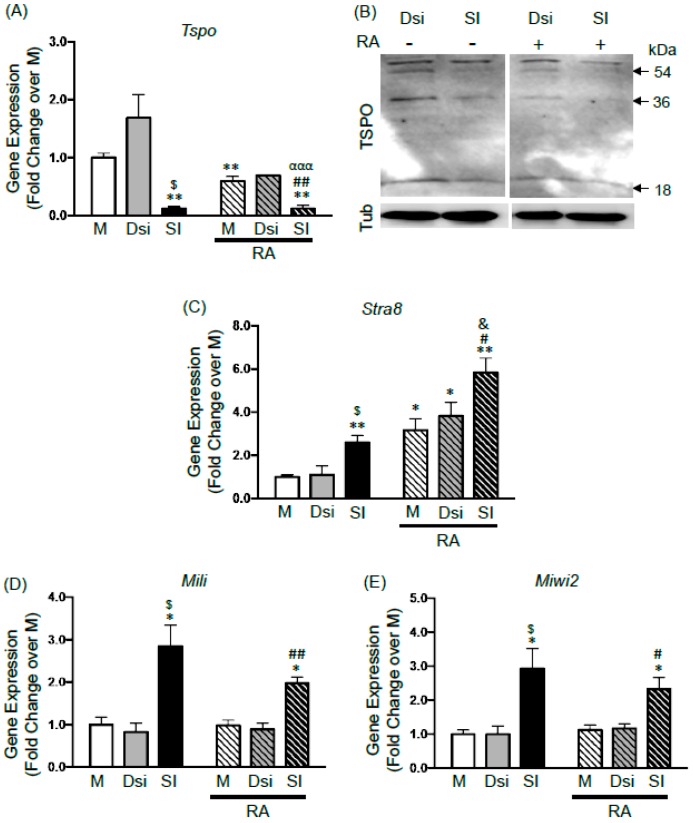
Effect of TSPO knockdown on gene expression in gonocytes. Silencing experiments were performed as described in the method section, by treating isolated gonocytes with either medium (mock; M), non-targeting DsiRNA (Dsi), or TSPO siRNA (SI) for 24 h (RNA analysis) or 48 h (protein analysis), followed by an additional 24 h incubation with or without RA (10^−6^ M). (**A**) *Tspo* mRNA expression. Results shown are from 3 independent germ cell preparations (each condition done in duplicate) and are plotted as mean ± SEM. * *p*-value < 0.05; and ** *p*-value < 0.01 compared to M; ^$^ *p*-value < 0.05 compared to Dsi; ^##^ *p*-value < 0.01 compared to M + RA; ^ααα^ *p* < 0.001 compared to Dsi + RA; (**B**) immunoblot analysis of TSPO protein expression. Representative gels are shown. TSPO monomers (18 kDa), dimers (36 kDa) and trimers (54 kDa) are indicated by arrows. Tubulin (Tub) = loading control; (**C**–**E**) mRNA expression of *Stra8* (**C**), *Mili* (**D**), and *Miwi2* (**E**). Results shown are from 3 independent germ cell preparations (each done in duplicate) expressed as means ± SEM. * *p*-value < 0.05; and ** *p*-value < 0.01 compared to M; ^$^ *p*-value < 0.05 compared to Dsi; ^#^ *p*-value < 0.05 and ^##^ *p*-value < 0.01 compared to M + RA; ^&^ *p*-value < 0.05 compared to SI.

To examine the potential role of TSPO in gonocyte differentiation, we knocked down its expression using siRNA transfection with Lipofectamine. Treatment with siRNA significantly reduced *Tspo* mRNA expression by 90% in isolated gonocytes after 24-h treatment, confirming knockdown (Figure 3A). As shown by immunoblot analysis, a successful TSPO knockdown was also seen at the protein level, affecting 18 kDa monomers, as well as 36 kDa dimers and 54 kDa trimers in cells treated with siRNA, compared to those treated with DsiRNA (Figure 3B), similar to mock samples (not shown).

RA-induced differentiation in mock and DsiRNA samples was confirmed by significant three- to four-fold increases in the mRNA expression of the differentiation marker *Stra8* [15,16,20] (Figure 3C). RA treatment significantly decreased *Tspo* mRNA expression by 40%, compared to levels found in control mock cells (Figure 3A); similarly to the results obtained with non-transfected gonocytes (Figure 1C). RA treatment also induced a decreasing trend in TSPO mRNA levels in cells treated with DsiRNA. Looking at protein levels, RA treatment decreased TSPO bands in DsiRNA—treated cells (Figure 3B). Furthermore, in siRNA knockdown cells, RA treatment for 24 h induced more pronounced decreases in TSPO protein bands than with siRNA alone (Figure 3B). Taken together, these results suggested that one of the effects of RA in differentiating gonocytes is to repress TSPO expression.

Next, we examined the effects of silencing TSPO expression on *Stra8* expression in gonocytes, in the absence and presence of RA. Interestingly, TSPO silencing induced a significant three-fold increase in *Stra8* mRNA levels in the absence of added RA (beyond the small RA amounts provided by 2.5% FBS), suggesting that removing TSPO promoted the expression of this differentiation gene in gonocytes (Figure 3C). The addition of 10^−6^ M RA in TSPO knockdown cells further doubled *Stra8* expression in comparison to its levels in TSPO knockdown cells and in control mock cells (Figure 3C). These data suggest that TSPO may play a repressive role in gonocyte differentiation, and that Stra8 is involved in removing TSPO to facilitate cell differentiation.

We then analyzed the mRNA expression profiles of *Mili* and *Miwi2*, two RNA binding proteins of the PIWI family critical for maintaining the genomic integrity of early germ cells, including gonocytes, by preventing transposon expression, and for the progression of spermatogenesis [21,22]. Interestingly, silencing TSPO significantly increased by three-fold the expression of both *Mili* and *Miwi2* transcripts (Figure 3E,F). RA treatment in mock, DsiRNA or TSPO knockdown cells had no effect on these genes, suggesting that these genes are not regulated by RA in differentiating gonocytes, while TSPO appears to play a repressive role in the expression of these genes.

### 2.4. TSPO Expression in Normal Human Adult Testicular Tissues

**Figure 4:**
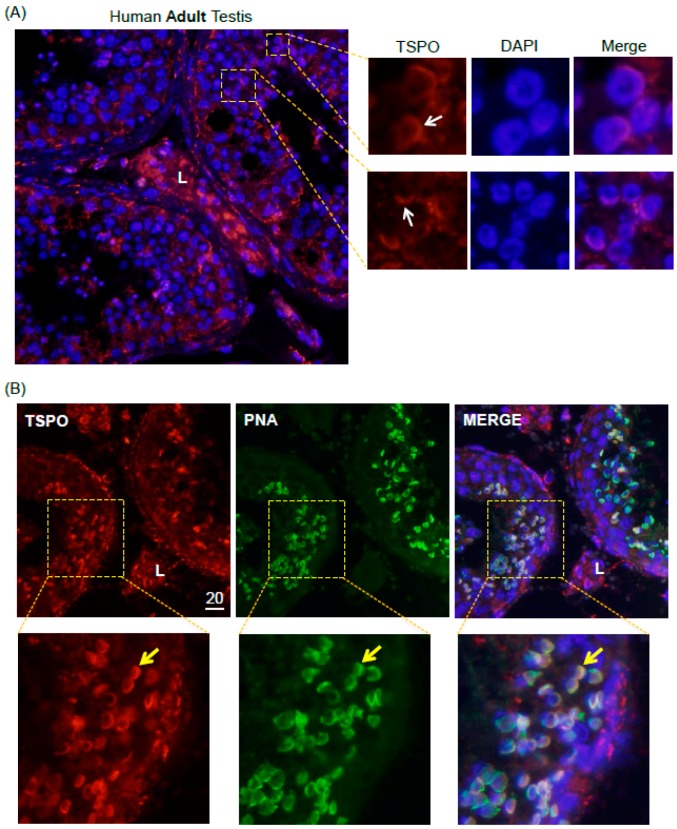
TSPO protein expression in normal human adult testis. Immunofluorescence (IF) analysis was performed on paraffin sections. (**A**) Patient 1. **Red**: TSPO protein expression; **Blue**: DAPI nuclear staining. Leydig cells are indicated by “L”. Areas showing expression in round spermatids (acrosomal formation) are shown in enlarged panels. Representative images are shown; (**B**) Patient 2. **Red**: TSPO protein expression; **Green**: PNA labeling; **Blue**: DAPI nuclear staining. Examples of co-localization between TSPO and PNA are indicated by yellow arrow in enlarged panels. Representative images are shown. Scale bar: 20 μm.

Considering TSPO expression in subsets of adult rat male germ cells, we next examined whether TSPO is also expressed in adult human spermatogenic cells. TSPO mRNA was examined by qPCR analysis in frozen biopsies from three patients with normal spermatogenesis, and TSPO protein expression were determined in testes paraffin sections from three other patients (Table 1). TSPO was highly expressed in the interstitial area, corresponding to its expected localization in Leydig cells (Figure 4A). Interestingly, TSPO was also expressed in round spermatids, more specifically, in what appeared to be the forming acrosomes (Figure 4A). To confirm that TSPO co-localized with nascent acrosomes, we performed immunofluorescence analysis on normal human adult testicular tissue biopsies using both an anti-TSPO antibody, and PNA, a peanut agglutinin lectin commonly used as acrosomal marker [23]. As shown in the magnified panels of Figure 4B, there was clear co-localization of TSPO and acrosomal immunofluorescent signals in distinct sections of tubules, indicating a stage-specific pattern of TSPO protein expression in human germ cells.

### 2.5. TSPO Expression in Seminomas

**Figure 5:**
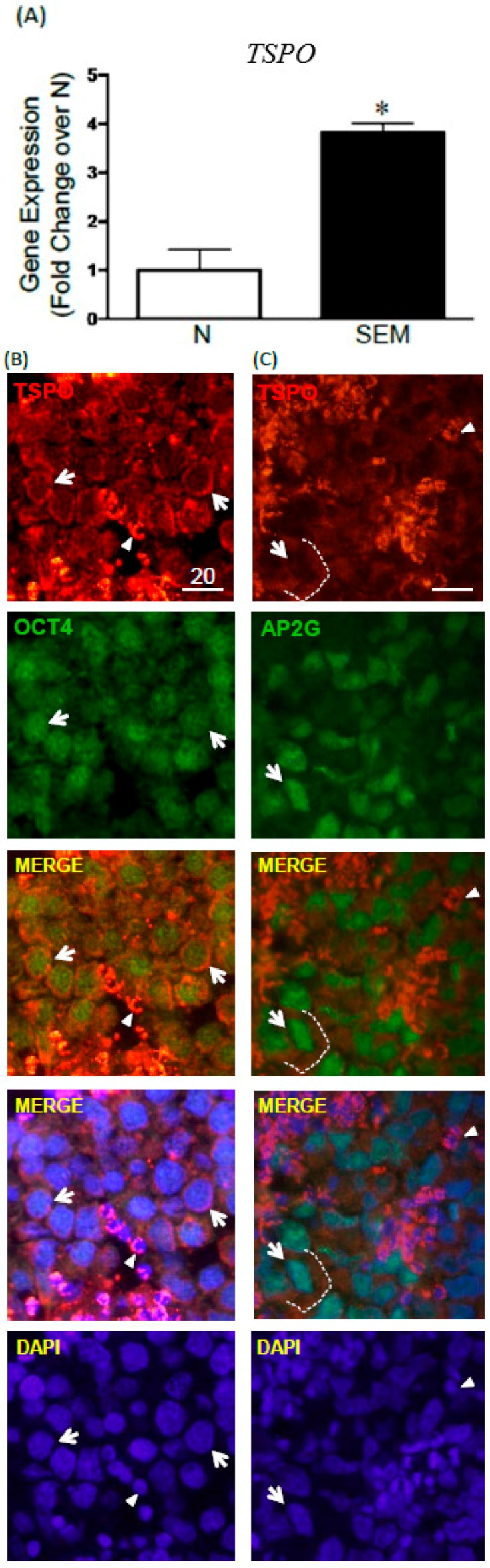
TSPO mRNA and protein expression in human seminomas. (**A**) *TSPO* mRNA expression in normal human adult testicular tissue and seminoma biopsies. *n* = 3–4, * *p*-value < 0.05; (**B**) TSPO (**red**) and OCT4 (**green**) protein expression in seminomas, visualized by IF in sections from Patient 4. Blue: DAPI nuclear staining. Representative images are shown; (**C**) TSPO (**red**) and AP2γ (**green**) protein expression in seminomas examined by IF in Patient 6. Representative images shown. (**B**,**C**) Arrows indicate representative seminoma cells; arrowheads indicate TSPO-positive small somatic cells (likely Leydig cells). Scale bars in Figure 4B,C are 20 μm.

Because of TSPO expression in gonocytes, and the suspected link between impaired gonocytes development and TGCT formation, we next examined whether TSPO might also be expressed in seminoma, the most common type of TGCTs. A comparison of *TSPO* transcript levels between normal testes and seminoma biopsies showed that seminomas significantly expressed higher levels of *TSPO* mRNA than normal testicular tissues (Figure 5A). TSPO protein expression was assessed in paraffin sections from several seminoma cases, in parallel with pluripotency genes OCT3/4 (OCT4; POU5F1) and AP2γ (TFAP2C), considered as seminoma markers [24,25]. Interestingly, TSPO was expressed in OCT4- and AP2G-positive cells, where it was localized in the cytoplasmic compartment, forming a ring around tumor cell nuclei, while the two transcription factors presented nuclear stainings (Figure 5B,C). The observation of specimen from different patients also highlighted zones of heterogeneity in some seminomas, with the presence of large cells with irregularly shaped nuclei, resembling embryonal carcinoma cells rather than seminoma cells (Figure 5C). In this case, TSPO appeared as a diffuse and weak signal spread around the nuclei, rather than as the well-defined cytoplasmic ring found in more rounded seminoma cells. In some specimens, a very strong TSPO signal was observed in OCT4-negative and AP2G-negative cells, much smaller in size than the tumor cells, likely corresponding to Leydig cells (Figure 5B,C). Taken together, these data showed that TSPO is expressed in seminoma cells, although at lower level than in the presumptive Leydig cells found within some tumors.

## 3. Discussion

TSPO is a ubiquitous and multifunctional protein [4,5,6,7], shown by various pharmacological and molecular means to play a critical role in steroidogenesis [26,27]. While TSPO main location in testis is in the mitochondria of steroidogenic Leydig cells, we have found in previous studies that TSPO is also expressed in rat and mouse male germ cells in an age and spermatogenic cycle dependent manner [8,9]. The goal of the present study was to examine if TSPO plays a role in gonocyte differentiation, an essential process at the basis of spermatogenesis, and to examine its patterns of expression in healthy human testicular germ cells and in seminomas, which are thought to arise from disrupted gonocyte differentiation [18].

Our initial finding that gonocytes express higher levels of TSPO than PND8 spermatogonia led us to speculate that TSPO expression is down-regulated during gonocyte differentiation. Moreover, the change in TSPO subcellular localization from nuclear in gonocytes to cytoplasmic in spermatogonia is reminiscent of other factors translocating in spermatogenic cells from one compartment to another in function of their activation status. For example, FOXO1 has been shown to translocate from cytoplasm to nucleus between PND1 and 3, a period corresponding to the transition from quiescence to mitosis, and to remain nuclear in SSC [28]. TSPO nuclear localization has been reported before, particularly in breast cancer cells, where it is involved in cell proliferation [4]. However, this does not seem to be the case in gonocytes, since adding a TSPO binding drug to gonocytes in presence or absence of proliferation factors did not have an effect [8].

To examine whether TSPO is down-regulated during gonocyte differentiation, isolated PND3 gonocytes were treated with the differentiation factor RA. Our data confirmed that RA exerts a repressive effect on the expression of TSPO mRNA and protein during gonocyte differentiation, a process characterized by an increase in Stra8 expression. Interestingly, TSPO promoter has been shown to contain putative binding sites for RARβ and RARγ [7]. Together with our previous data showing that PND3 gonocytes express high levels of RARγ [16], this suggests that RARγ activation may be involved in TSPO down-regulation.

Similar effects were observed in F9 mouse embryonal carcinoma cells, a cell line issued from a mouse testicular teratoma, which shares a number of pluripotency markers with embryonic stem cells as well as gonocytes [15,16,29,30]. In F9 cells, which express genes from both the somatic and germline lineages, RA treatment can induce the expression of markers of somatic (e.g., collagen IV) and germ cell (e.g., Stra8) fates, depending of the culture conditions and downstream pathway activated [16,30]. All TGCTs, including embryonal carcinoma, are believed to derive from a common precursor, the carcinoma in situ, itself resulting from the failed differentiation of gonocytes [18]. Therefore, one cannot exclude at present that RA-induced TSPO repression in F9 cells may correspond to a function retained from gonocytes, which was not altered during the process of carcinogenesis.

To better understand TSPO role in gonocyte differentiation, we knocked down *Tspo* in gonocytes treated or not with RA. Surprisingly, silencing TSPO expression with siRNA was sufficient to significantly increase *Stra8* expression in gonocytes, in basal conditions with low levels of RA provided by FBS. Moreover, TSPO knock down potentiated RA-mediated increases in *Stra8* transcripts, implying that TSPO represses Stra8 expression. Among its multiple roles, TSPO has been reported to be positively regulated during adipocyte differentiation and homeostasis, with its knockdown impairing the release of adipokines, glucose uptake, and adipogenesis [31,32]. We have previously shown that gonocyte differentiation involves the activation of platelet-derived growth factor receptors and downstream SRC, JAK2, and STAT5 pathways [16] and an active ubiquitin proteasome system [20]. In contrast to these positive regulators of gonocyte differentiation, the present results suggest that TSPO acts as a repressor on gonocyte differentiation, and that one of RA effects is to eliminate this block. This is reminiscent of the downregulation by RA of Nanos2 in fetal and neonatal gonocytes, where this RNA binding protein was shown to repress Stra8 expression and prevent premature entry of germ cells into meiosis [33]. It would be interesting to examine in future studies whether TSPO ligands with agonist properties might exert a repressing effect on gonocyte differentiation, as suggested by our silencing results.

We then analyzed the mRNA expression of two germ cell markers, the PIWI-interacting RNA binding proteins Mili, and Miwi2, both required for spermatogenesis and meiotic progression. MILI is expressed from gonocytes to early spermatocytes, and has been shown to be essential for spermatogonial stem cell self-renewal and differentiation [21]. MIWI2 is strongly expressed in gonocytes, where it is guiding the re-methylation of transposons and preventing their activation, following the global erasure of DNA methylation marks in primordial germ cells [22]. Besides their role in transposon methylation, these genes have also been implicated in histone modifications and post-transcriptional regulation [34]. While the expression patterns of the two genes did not appear to be regulated by RA, TSPO knockdown led to increases in both *Mili* and *Miwi2* transcripts, suggesting TSPO involvement in the down-regulation of these genes, independent of differentiation signals. While preserving the genomic integrity of germ cells is critical for species survival, allowing for some degree of transposon mobility has played a role in evolution and adaptability of species to environmental changes. Considering the multiple roles of TSPO, the fact that it is highly conserved throughout evolution from bacteria to human [35], and its nuclear localization in gonocytes, one could envision a role for TSPO in maintaining the balance between transposon mobility and preserving genomic integrity in gonocytes. Further experiments will be required to test this hypothesis.

Next, we examined whether TSPO is also expressed in human normal and pathological germ cells, which had not been reported in the literature. Our study showed that normal testicular tissues express TSPO in discreet phases of spermatogenic cells, similarly to our previous findings in rat and mouse testis [8,9]. In human testis, besides its expected high expression in Leydig cells, TSPO was clearly expressed in the forming acrosomes of round spermatids, as confirmed by co-localization with PNA, a peanut agglutinin lectin, commonly used as an acrosomal marker [23]. This is a novel finding, suggesting that TSPO is involved in the formation of specialized organelles in germ cells. Interestingly, TSPO has been previously reported to be expressed in the Golgi apparatus of liver cells, suggesting that it might play a similar role in somatic cells [36].

Finally, in our search of TSPO role in germ cells, we examined its expression patterns in seminoma, the most common testicular germ cell tumor type in human, proposed to originate from abnormal gonocytes [18]. The comparative study of several normal and seminoma samples revealed a significant upregulation in *TSPO* mRNA levels in seminomas. Immunofluorescent analysis of TSPO and two pluripotency transcription factors OCT3/4 and AP2γ used as seminoma markers [24,25] confirmed TSPO protein localization in seminoma cells. Several studies have reported changes in TSPO expression and potential role in various cancers. For example, TSPO expression is upregulated in glioma tumor cells, compared to normal brain [37]. TSPO has also been implicated in lung cancer development, as cigarette smoke exposure was found to alter TSPO protein, leading the way for the initiation and progression of lung cancer [38]. Studies have shown that TSPO is highly expressed in estrogen-receptor (ER) negative breast tumors, representing a potential target for the development of new therapies for this subset of breast cancer [39]. Moreover, studies have shown that that TSPO expression correlates positively with the invasiveness and/or malignancy of breast, colorectal and prostate cancers [4,40,41,42]. Thus, our study revealed one more type of cancer presenting TSPO dysregulation. Determining whether TSPO alterations reflect its involvement in testicular tumorigenesis process, or if they indicate the retention of an early germ cell marker in deficient germ cells remains to be determined.

Taken together, our study shows that TSPO is down regulated during gonocyte differentiation, in which it might play a repressive role. Moreover, our expression studies in human normal testes confirmed that TSPO is expressed in subsets of adult germ cells, suggesting a function in acrosome formation, while the analysis of tumor samples revealed its mRNA up-regulation and protein localization in seminoma cells. Further studies are needed to determine the exact role of TSPO in normal spermatogenic cell development, from gonocyte to more mature germ cells, and in testicular cancer.

## 4. Materials and Methods

### 4.1. Animals

Newborn male Sprague Dawley rats were obtained from Charles Rivers Laboratories (Saint-Constant, QC, Canada). PND3 and PND8 pups were euthanized and handled according to protocols approved by the McGill University Health Centre Animal Care Committee and the Canadian Council on Animal Care (McGill Animal Care protocol #2015-7582; approved July 2015).

### 4.2. Germ Cell Isolation

Gonocytes were isolated from PND3 rat testes as previously described [13,43]. Briefly, testes from 40 rats were isolated and decapsulated. Gonocytes were isolated by sequential enzymatic digestion and differential plating overnight in RPMI 1640 medium (Invitrogen, Burlington, ON, Canada) with 5% fetal bovine serum (FBS) (Invitrogen), 2% penicillin/streptomycin (CellGro, Manassas, VA, USA), and 1% amphotericin B (CellGro). The next morning, non-adherent germ cells were further separated using a 2%–4% bovine serum albumin (BSA) (Roche Diagnostics, Indianapolis, IN, USA) gradient. Gonocytes were identified by morphology and by their larger size compared to Sertoli/myoid cells. Fractions containing the most gonocytes were pooled, centrifuged, and collected with a purity of at least 85% for RNA analysis. Samples with lower purity were used for immunocytochemical analysis. Spermatogonia were isolated from the testes of 10 PND8 pups per preparation, using the same method used for gonocyte isolation described above [20,43]. All experiments were performed using a minimum of three independent gonocyte or spermatogonia preparations.

### 4.3. Gonocyte Treatment

Isolated gonocytes were cultured in RPMI media (Invitrogen) containing 2.5% fetal bovine serum (FBS), 2% penicillin/streptomycin (CellGro) and 1% amphotericin B (CellGro) at 37 °C and 3.5% CO_2_. Gonocytes were plated at a density of 8000–10,000 cells/well in 24-well plates. The cells were treated with or without all-trans retinoic acid (RA) (10^−6^ M) (Sigma, Oakville, ON, Canada) for 24 h (RNA analysis) or 72 h (protein analysis). Within each experiment, duplicate wells were used for each treatment condition.

### 4.4. TSPO Knockdown Using Silencing RNA

For silencing experiments, once gonocytes were collected from the BSA gradient, they were transfected with 100 nM of a mixture of three pre-designed siRNA sequences (Table 2) (IDT, San Jose, CA, USA) using Lipofectamine RNAiMAX (Invitrogen) and Opti-MEM transfection medium (Invitrogen). RPMI culture media without antibiotics, FBS or amphotericin B was used to plate the cells. A red fluorescent dye and a non-targeting universal negative control DsiRNA were transfected at 10 nM each, and served as positive and negative controls, respectively (IDT). Cells were transfected for 24 h (mRNA silencing analysis) and 48 h (protein silencing analysis), and then treated with or without RA (10^−6^ M) (Sigma) for an additional 24 h, in medium supplemented with 2.5% FBS.

### 4.5. F9 Mouse Embryonal Carcinoma Cell Culture

F9 cells were maintained in DMEM medium (Invitrogen) containing 10% FBS (Invitrogen) at 37 °C and 5.0% CO_2_. Cells were plated on gelatine-coated culture dishes on day 1 and treated on day 2 with or without RA (10^−7^ M) (Sigma) for 24 h and 72 h for mRNA and protein analysis, respectively.

### 4.6. Human Specimens

Frozen and paraffin-embedded tissues from human adult normal testes and seminoma specimens, together with their pathology reports, were obtained from the Departments of Pathology and Surgery at the McGill University Health Centre, Montreal, and at La Paz University Hospital (Universidad Autonoma de Madrid, Madrid, Spain), with informed consent forms, following institutional ethics review board requirements, as described before [44]. Six non-cancer samples presenting normal seminiferous tubules and spermatogenesis, as well as seven seminoma specimens were examined (see Table 1 for details).

### 4.7. RNA Extraction and cDNA Synthesis

Total RNA was extracted from cell pellets or frozen biopsies using the PicoPure RNA isolation kit (Arcturus, Mountain View, CA, USA) and digested with DNase I (Qiagen, Valencia, CA, USA). For quantitative PCR (qPCR) analysis, cDNA was synthesized from the extracted RNA by using the single strand cDNA transcriptor synthesis kit (Roche Diagnostics) following the manufacturer’s instructions.

### 4.8. Quantitative Real Time PCR (qPCR)

QPCR was performed using a LightCycler 480 with a SYBR Green PCR Master Mix kit (Roche Diagnostics). The primer sets used were designed using the Roche primer design software (Roche Diagnostics) and are listed in Table 3. QPCR cycling conditions were as follows: Initial step at 95 °C followed by 45 cycles at 95 °C for 10 s, 61 °C for 10 s, and 72 °C for 10 s. The direct detection of PCR products was monitored by measuring the increase in fluorescence caused by the binding of SYBR Green dye to double-stranded DNA. The comparative threshold cycle (*C*_t_) method was used to analyze the data and 18S rRNA was used for data normalization, as it was previously shown to be an adequate housekeeping gene for both rat germ cells and human seminomas [20,44]. Assays were performed in triplicate. All experiments were performed using a minimum of three independent sample preparations, with each condition performed in duplicate, and the mean ± SEM are shown.

### 4.9. Immunoblot Analysis

F9 cells or gonocytes were solubilized in Laemmli buffer and protein concentrations were determined using the BCA protein assay kit (Thermo Scientific, Burlington, ON, Canada). As previously described [20], proteins (30 μg per lane) were separated on pre-casted 4%–20% tris-glycine gels (Invitrogen). Gels were transferred to a polyvinylidene fluoride (PVDF) membrane (Bio-Rad, Mississauga, ON, Canada). After blocking with 5% milk, membranes were probed with an anti TSPO antibody (lab-made; [8]) to determine protein expression. GAPDH and tubulin (Trevigen, Gaithersburg, MD, USA) were used as loading controls. After an overnight incubation at 4 °C, bound antibodies were detected using anti-biotin and horseradish peroxidase (HRP) coupled goat anti-rabbit secondary antibodies (Cell Signaling, Danvers, MA, USA) and ECL-enhanced chemiluminescence (GE Healthcare, Mississauga, ON, Canada). Images were taken using the LAS-4000 gel documentation system (Fujifilm, Mississauga, ON, Canada).

### 4.10. Immunocytochemistry (ICC)

Microscopic slides were prepared using isolated germ cells and treated gonocytes collected by cytospin centrifugation, and processed for ICC as previously described [20,43]. In brief, slides were treated with Dako Target Retrieval solution (DAKO, Burlington, ON, Canada), a 30% hydrogen peroxide methanol solution, and then non-specific protein interactions were blocked with PBS (Invitrogen) containing 10% goat serum (Vector Laboratories, Burlington, ON, Canada), 1% BSA (Roche Diagnostics) and 0.02% Triton X100 (Promega; Madison, WI, USA) for one hour; and then incubated with anti TSPO primary antibody (lab-made) diluted in PBS containing 1% BSA (Roche Diagnostics) and 0.02% Triton X100 (Promega) overnight at 4 °C. The slides were then incubated with a biotin-conjugated goat anti-rabbit secondary antibody (BD Pharmingen, San Diego, CA, USA) diluted in PBS-1% BSA for one hour at room temperature. Immunoreactivity was detected using a combination of streptavidin-peroxidase (Invitrogen) and AEC single use solution (Invitrogen). Slides were counter-stained with hematoxylin (Sigma Aldrich), coated with Crystal Mount (Electron Microscopy Sciences, Hatfield, PA, USA) and dried, and then cover-slipped using Permaflour (Thermo Scientific) and glass coverslips (Fisher Scientific, Toronto, ON, Canada). The slides were then viewed using a BX40 Olympus microscope (Olympus, Center Valley, PA, USA) coupled to a DP70 Olympus digital camera (Olympus).

### 4.11. Immunofluorescence (IF)

Paraffin-embedded human adult normal testicular tissues and seminoma specimens were processed for IF as previously described [44], following a protocol similar to that in the ICC section above, except that slides were dewaxed and rehydrated using Citrosolv (Fisher Scientific) and Trilogy solution (Cell Marque IVD, Rocklin, CA, USA), prior treatment with Dako Target Retrieval solution. Sections were incubated overnight with a combination of either TSPO antibody (Epitomics, Burlingame, CA, USA) and OCT 3/4 antibody (Santa Cruz, Dallas, TX, USA) diluted in PBS containing 1% BSA and 0.02% Triton X100 overnight at 4 °C, or TSPO antibody and AP2γ antibody (Santa Cruz), or TSPO antibody alone. Next, slides were incubated with a fluorescent goat anti-rabbit Alexa Fluor 546 secondary antibody and a fluorescent goat anti-mouse Alexa Fluor 488 secondary antibody (Invitrogen) diluted in PBS (Invitrogen) containing 1% BSA (Roche Diagnostics) for one hour at room temperature. Some slides were then stained for 30 min with a lectin from *Arachis hypogaea* (peanut) PNA (Sigma) to stain the acrosomes. Nuclear staining for all slides was done using nuclear DAPI (Invitrogen) staining for 5 min. Slides were mounted with PermaFluor Mountant (Thermo Scientific) and cover-slipped. Slides were then examined using a BX40 Olympus microscope (Olympus) coupled to a DP70 Olympus digital camera (Olympus).

### 4.12. Statistical Analysis

Statistical analysis was performed using an unpaired two-tail Student’s *t*-test using statistical analysis functions in the GraphPad Prism 5.0 program (GraphPad Inc., San Diego, CA, USA). All experiments were performed with a minimum of three independent experiments, each including duplicate wells per condition. A *p*-value less than 0.05 was considered statistically significant.

## Figures and Tables

**Table 1:** Human normal and tumoral testicular tissue information.

Patient	Pathology	Age	Tissue Type
Samples for qPCR analysis			
1	Normal		Normal spermatogenesis, Vasectomy patient
2	Normal		Normal spermatogenesis, Vasectomy patient
3	Normal		Normal spermatogenesis, Vasectomy patient
4	Seminoma		Left testis, Seminoma, Age 36, Stage I
5	Seminoma		Right testis, Seminoma, Age 26, Stage I
6	Seminoma		Right testis, Seminoma, Age 50, Stage I
Samples for IF analysis			
1	Normal	73	Normal testicular parenchyma
2	Normal	57	Normal testicular parenchyma
3	Normal	73	Normal testicular parenchyma
4	Seminoma	NA	100% Seminoma
5	Seminoma	NA	100% Seminoma
6	Seminoma	38	100% Seminoma
7	Seminoma	NA	100% Seminoma

**Table 2:** Translocator protein 18 kDa (TSPO) siRNA sequences. Three siRNA pairs (#1–3) were used.

#	Sense Strand	Antisense Strand
1	5′-CCUACAUAAUCUGGAAAGAGCUGGG-3	5′-CCCAGCUCUUUCCAGAUUAUGUAGGAG-3′
2	5′-GCAGUUGCAAUCACUAUGUCUCAAT-3′	5′-AUUGAGACAUAGUGAUUGCAACUGCUG-3′
3	5′-CACCUAGCCAUCAGGAAUGCAGCCC-3′	5′-AUUGAGACAUAGUGAUUGCAACUGCUG-3′

**Table 3:** Quantitative real time PCR primers. Lower case character letters in sequences were added by the primer designing program and are not part of the gene sequences.

Species	Gene	Forward Sequence	Reverse Sequence
Rat	*Tspo*	cgcaATGGGAGCCTACTTTGTGcG	GCCAGGAGGGTTTCTGCAAG
Rat	*Stra8*	TGCTTTTGATGTGGCGAGCT	GCGCTGATGTTAGACAGACGCT
Rat	*Mili*	cggaaGACATCCAGTACAGAGTCATTCcG	GGGTCTCTTCTTGCTCGCTGA
Rat	*Miwi2*	AGCTGCACAGGTATTCCGCTAGAA	ATGGCAGACAGGACTTCGTCGATT
Rat	*18s*	cgggTGCTCTTAGCTGAGTGTCCcG	CTCGGGCCTGCTTTGAACAC
Mouse	*Tspo*	CCCGCTTGCTGTACCCTTACC	CACCGCATACATAGTAGTTGAGCAC
Mouse	*18s*	CGGAATCTTAATCATGGCCTCAGTTC	ACCGCAGCTAGGAATAATGGAAT
Human	*TSPO*	GGTGGATCTCCTGCTGGTCA	CGACCAATACGCAGTAGTTGAGTGTG
Human	*18S*	cggacTGTGATGCCCTTAGATGTCcG	GTAGGGTAGGCACACGCTGAG
